# Efficacy of Gradient Compression Garments in the Hours After Long-Duration Spaceflight

**DOI:** 10.3389/fphys.2020.00784

**Published:** 2020-07-17

**Authors:** Stuart M. C. Lee, L. Christine Ribeiro, Steven S. Laurie, Alan H. Feiveson, Vladimir V. Kitov, Igor S. Kofman, Brandon R. Macias, Marissa Rosenberg, Ilya V. Rukavishnikov, Elena S. Tomilovskaya, Jacob J. Bloomberg, Inessa B. Kozlovskaya, Millard F. Reschke, Michael B. Stenger

**Affiliations:** ^1^ KBR, Houston, TX, United States; ^2^ Lyndon B. Johnson Space Center, National Aeronautics and Space Administration, Houston, TX, United States; ^3^ Department of Sensory-Motor Physiology and Countermeasures, Institute of Biomedical Problems of the Russian Academy of Sciences, Moscow, Russia

**Keywords:** stand test, orthostatic tolerance, lower body compression, heart rate, blood pressure, International Space Station

## Abstract

The incidence of presyncopal events is high soon after a long-duration spaceflight;>60% of returning astronauts could not complete a 10-min 80° head-up tilt test on landing day (R+0) after ~6 months of spaceflight. The objective of this study was to demonstrate the ability of a lower body gradient compression garment (GCG) to protect against an excessive increase in heart rate and a decrease in blood pressure during standing after long-duration spaceflight.

**Methods**: Eleven astronauts (9 M, 2 F) volunteered to participate. The stand test protocol consisted of 2 min of prone rest followed by 3.5 min of standing. Subjects completed one familiarization session, two preflight data collection sessions in standard clothing, and three tests on landing day while wearing GCG. Postflight tests were conducted 1–4 h (R+0A), ~12 h (R+0B), and ~28 h after landing (R+0C).

**Results**: All astronauts completed the stand test preflight. Three astronauts were unable to attempt the stand test at R+0A, and one of these was unable to start the test at R+0B. One astronaut was unable to complete 3.5 min of standing at R+0B (test ended at 3.3 min). Review of the individual’s blood pressure data revealed no hypotension but the astronaut reported significant motion sickness. Of the astronauts who participated in testing on landing day, the heart rate and mean arterial pressure responses to standing (stand-prone) were not different than preflight at any of the postflight sessions.

**Conclusion**: Wearing the GCG after spaceflight prevented the tachycardia that normally occurs while standing after spaceflight without compression garments and protected against a decrease in blood pressure during a short stand test.

## Introduction

We have previously reported that 60–80% of astronauts experienced orthostatic intolerance during 10 min of 80° head-up tilt conducted in the controlled conditions of the laboratory 4–6 h after landing from long-duration spaceflight (Meck et al., 2001; Lee et al., 2015). Currently, astronauts returning from the International Space Station (ISS) under normal circumstances receive assistance from ground support personnel to exit the Soyuz vehicle and are attended to by medical personnel immediately after landing (Baker et al., 2019), thus controlling the risk of orthostatic intolerance. However, in off-nominal conditions, such as a ballistic re-entry when the Soyuz capsule lands miles from support personnel (Carreau, 2008; Pettit, 2010), the crew must function autonomously, and thus the consequences of the astronauts experiencing orthostatic intolerance may be more substantial. Therefore, instituting countermeasures that reduce the likelihood of orthostatic intolerance to preserve capabilities in the minutes and hours after landing can be critical for astronaut health and safety.

We have studied cardiovascular responses to standing and during the performance of functional tasks following Space Shuttle (Arzeno et al., 2013; Stenger et al., 2013) and earlier ISS missions (Mulavara et al., 2018) without lower body compression garments, but postflight testing of ISS astronauts was not possible in the previous study until ~24 h after landing. However, recovery of the orthostatic responses to upright posture is profound within the first 24 h of landing. There are anecdotal reports of signs and symptoms of orthostatic intolerance at the landing site (Pettit, 2010), but the incidence of presyncope during tilt tests is substantially reduced the day after landing (Lee et al., 2015). Thus, the objective of this study was to quantify the effectiveness of the gradient compression garment (GCG) immediately post-landing and during the first day of recovery. We have previously demonstrated the efficacy of a next-generation lower body GCG after 2 weeks of bed rest as a spaceflight analog (Stenger et al., 2014) and during the first few hours after Space Shuttle missions (Stenger et al., 2013), but it was unclear as to whether these garments would provide sufficient protection from orthostatic intolerance immediately after landing from longer missions. Heart rate and blood pressure measurements during preflight stand tests without the GCG were compared to those obtained when astronauts completed the same testing protocol three times in the first 24 h after landing while wearing the GCG.

## Materials and Methods

This was a joint study conducted by the Cardiovascular and Vision Laboratory and the Neurosciences Laboratory at National Aeronautics and Space Administration (NASA) Johnson Space Center in collaboration with the Institute of Biomedical Problems of the Russian Academy of Sciences. Eleven astronauts from NASA, the European Space Agency, and the Japan Aerospace Exploration Agency (9 M, 2 F; 50 ± 6 year; 77.8 ± 8.7 kg; 173.0 ± 5.9 cm; 26.0 ± 3.3 kg·m^−2^; mean ± SD), who completed ISS missions (194 ± 65 day) consented to participate in this investigation. Study protocols and procedures were reviewed and approved by the NASA Johnson Space Center Institutional Review Board, Institute of Biomedical Problems Bioethics Committee, and the ISS Human Research Multilateral Review Board.

### Data Collection Timeline

Astronauts completed preflight testing in normal clothing in the laboratory. Astronauts participated in a familiarization session scheduled ~180 days before launch and two preflight data collections at ~90 (L-90) and ~60 days (L-60) days before launch. All astronauts landed in the Russian Soyuz spacecraft, and therefore all were required to wear the Russian lower body compression garment (Kentavr) during re-entry and landing (Vil-Viliams et al., 1998; Platts et al., 2009). After extraction from the capsule at the landing zone, astronauts were carried to the medical tent for a brief physical examination by their flight surgeon. Thereafter, with the assistance of trained operators and the crew surgeon, astronauts doffed their Kentavr and donned the GCG. Testing occurred as soon as possible after landing (R+0A), either in the tent at the Soyuz landing site or after transport by helicopter to Karaganda or Dzhezkazgan Airports in Kazakhstan. No intravenous fluids (IV) were administered prior to testing at the landing site, but often astronauts received at least 1 L of IV fluids during transport by helicopter to the airport (Mulavara et al., 2018). The number of astronauts participating at each of the postflight test sessions varied. At R+0A, six of the seven astronauts participated in testing in the tent (1.9 ± 0.7 h after landing; range: 1.2–2.6 h), and one completed testing at the airport (4.3 h after landing). Subsequent testing (R+0B) occurred 12.2 ± 1.0 h after landing (range: 10.7–13.9 h) either in Germany (*n* = 1), Norway (*n* = 4), or Scotland (*n* = 4) at the refueling stop during travel back to Houston and again at Johnson Space Center (R+0C) 27.7 ± 1.8 h after the landing (range: 25.6–30.7 h, *n* = 11). Flight surgeons were requested to maintain a log of fluids and food consumed as well as medication and IV administered from the time of landing until R+0C. Subjects were not required to wear the GCG between test sessions.

### Stand Test

Subjects were instrumented with a Holter monitor (Mortara H12+, Mortara Instruments, Milwaukee, WI) for continuous recording of ECG (1 kHz) and calculation of heart rate, a Portapres® ambulatory blood pressure monitor (Finapres Medical Systems B.V., The Netherlands) for the continuous recording of arterial blood pressure (100 Hz) with height correction, and a calibrated blood pressure sphygmomanometer and brachial cuff (Welch Allyn, Skaneateles Falls, NY).

The stand test began with the astronaut prone on a mat. A manual blood pressure measurement was obtained by ~1 min and 45 s of rest, and then the astronaut stood as quickly as possible when a command to stand was issued at 2 min. Astronauts were instructed to not press down on the finger cuff while standing up from prone so as not to disturb the blood pressure signal. Astronauts stood with their gaze facing forward for 3.5 min. At the completion of the stand test, the test operator asked the astronaut to report their perception of motion sickness, ranging from 1 (no symptoms) to 20 (nausea to the point of vomiting).

For preflight testing in particular, astronauts were asked to maintain normal behavior patterns for intake of alcohol or caffeine prior to testing, maintain normal medications, avoid exposure to unusual motion conditions such as NASA’s Neutral Buoyancy Laboratory training or virtual reality training for at least 24 h, and avoid maximal exercise in the 24 h before testing. For all tests, astronauts were requested to avoid alcohol consumption, exercise, and heavy meals within 4 h before the session (light snack within 2 h prior to testing was acceptable).

### Gradient Compression Garment

In collaboration with the manufacturer of JOBST medical compression garments (Essity, Stockholm, Sweden), we developed an elastic three-piece GCG consisting of two thigh-high stockings and shorts that extend to the bottom of the rib cage that provides a continuous gradient of compression from the feet to the top of the garment. Compression is 55 mmHg at the ankle and gradually decreases along the leg to 35 mmHg at the knee and 18 mmHg at the top of the thigh, and further reduces to ~16 mmHg compression over the abdomen.

The GCGs were constructed for each subject based on detailed abdominal and lower body circumferences measured approximately 120 days before launch (L-120). Leg circumference was measured every 3.8 cm (1.5 inch) from the base of the toes to the top of the thigh. Additional measurements were obtained along the torso ending just below the breast-line. The desired tension was verified by the manufacturer when the garments were stretched to dimensions similar to that expected when the subjects donned the GCG using a Hosiery and Allied Trades Research Association (HATRA) test instrument that is identified in the British Standard for testing compression in elastic stockings. Validation of this line of garments was reviewed and approved by the FDA. Subjects donned the garments at L-90 to verify proper sizing and comfort. Due to time constraints on landing day, we were unable to measure the level of compression during postflight testing.

### End-of-Mission Fluid Loading

Astronauts are advised by Russian medical specialists to consume 18–20 ml/kg body weight of sodium chloride-water solution or equivalent dry salt with water with 3–4 meals in the last 12–20 h before landing (Kozlovskaya et al., 1995). Kozlovskaya and Grigoriev (2004) report that cosmonauts who participate in this form of end-of-mission fluid loading better tolerate the final phase of the spaceflight mission and the postflight reconditioning program.

### Data Reduction

Ectopic beats and artifacts were removed from the R-wave to R-wave (R-R) tracing derived from the Holter monitor and from the continuous blood pressure tracing before analysis through visual inspection (Arzeno et al., 2013). R-R and blood pressure data during the transition from prone to standing were not included in the analyses; blood pressure and electrocardiogram data from the time that the subject was fully upright until the time that the data were stable were discarded. Data were considered stable following the brief decrease in blood pressure with the transition to standing that sometimes was difficult to interpret due to artifact from the astronauts pressing on the finger cuff when pushing off the floor. The transition time from prone to standing was not consistent across test days, likely related to postflight sensorimotor disturbances, instability, and decreased muscle strength (L-90: 4.8 ± 1.7 s, range 2.7–9.4 s, *n* = 11; L-60: 4.8 ± 2.4 s, range 2.6–11.5 s, *n* = 11; R+0A: 14.8 ± 3.5 s, range 11.1–20.0 s, *n* = 7; R+0B: 10.8 ± 4.7 s, range 6.7–20.3, *n* = 9; R+0C: 8.2 ± 2.3 s, range 4.4–13.0 s, *n* = 11), as has been previously observed (Miller et al., 2018; Mulavara et al., 2018). Mean heart rate and mean arterial pressure were calculated for the 2 min of prone rest and ~3 min of standing. The manual blood pressure obtained during the prone period was used to calibrate the Portapres blood pressure signal to the manual blood pressure.

### Statistical Analyses

Data from all the astronauts were available from the preflight testing, but results from the familiarization session on L-180 were not used in these analyses. Data collection in the field environment precluded acquisition of all data in the postflight period such that calibrated blood pressure data were not available for two subjects and during standing for one subject at R+0B. Thus, these data were not considered in our analyses. Data from the one astronaut who stood for all but the last 12 s of the standing on R+0B were included in these analyses.

Taking into account the longitudinal design and missing data, mixed linear regression models with random intercepts at the subject level and fixed session effects were used to estimate mean heart rate, mean arterial pressure, and the response to standing (stand-prone) for heart rate (ΔHR) and mean arterial pressure (ΔMAP) for preflight and each of three postflight test sessions as well as the change from preflight for each postflight session. ΔHR and ΔMAP were analyzed separately for each combination of posture (prone and standing). In all analyses, standard errors were estimated by clustered bootstrapping to account for non-normality of residuals. After fitting the ΔHR regression models, point estimates and 95% confidence intervals for the mean preflight to postflight change in heart rate, mean arterial pressure, ΔHR, and ΔMAP were calculated to provide a quantitative assessment of how well compression garments can control the amount of these changes during recovery from spaceflight, taking uncertainty into account. Following the approach of Fraser (2019), we also report *p*-value function plots that express the relative support of the data for preflight to postflight changes in ΔHR mean exceeding or changes in ΔMAP mean lower than hypothetical values within a range of interest.

Given that there was no control group of ISS astronauts for this study, we compared preflight to postflight changes in ΔHR from a similar study without compression garments (Mulavara et al., 2018) to those observed in the current study using a mixed regression model accommodating study-specific between-subject variances on combined data on both studies. In the comparison subjects, ISS astronauts (11 M, 2 F) participated in inflight exercise countermeasures as prescribed by specialists from their respective space agency using a combination of resistive and cardiovascular exercise (Loehr et al., 2015), completed 159 ± 17 day of spaceflight, and were tested ~1 day after landing, similar to R+0C. Bed rest subjects (9 M) participated in an exercise countermeasure protocol similar to that used by ISS astronauts, did not participate in an end-of-bed rest fluid loading protocol, and were tested within an hour of rising from 70 days of 6° head-down tilt bed rest, similar to R+0A. Formal statistical inference on the effectiveness of the compression garments was based on this comparison for the outcome ΔHR at R+0A and at R+0C with significance defined as *p* < 0.025, controlling the family-wise Type I error to 0.05 or less (Bonferroni adjustment). No data corresponding to R+0B were available.

## Results

All astronauts were able to complete all the tests before flight, and all participated in at least one test on landing day. Data from one subject at R+0A and R+0B were not analyzed because the astronaut was provided the wrong size GCG for those tests, but this individual did wear the correct GCG at R+0C so those data are included in the analyses. Of the remaining 10 astronauts, three were unable to participate in the stand test at R+0A, and one of these also was unable to participate in the stand test at R+0B. Of the astronauts who participated in the stand test on landing day, the mean motion sickness score was 10 (range: 3–18), 6 (range: 2–14), and 5 (range: 1–13) at R+0A, R+0B, and R+0C, respectively.

**Figure 1:**
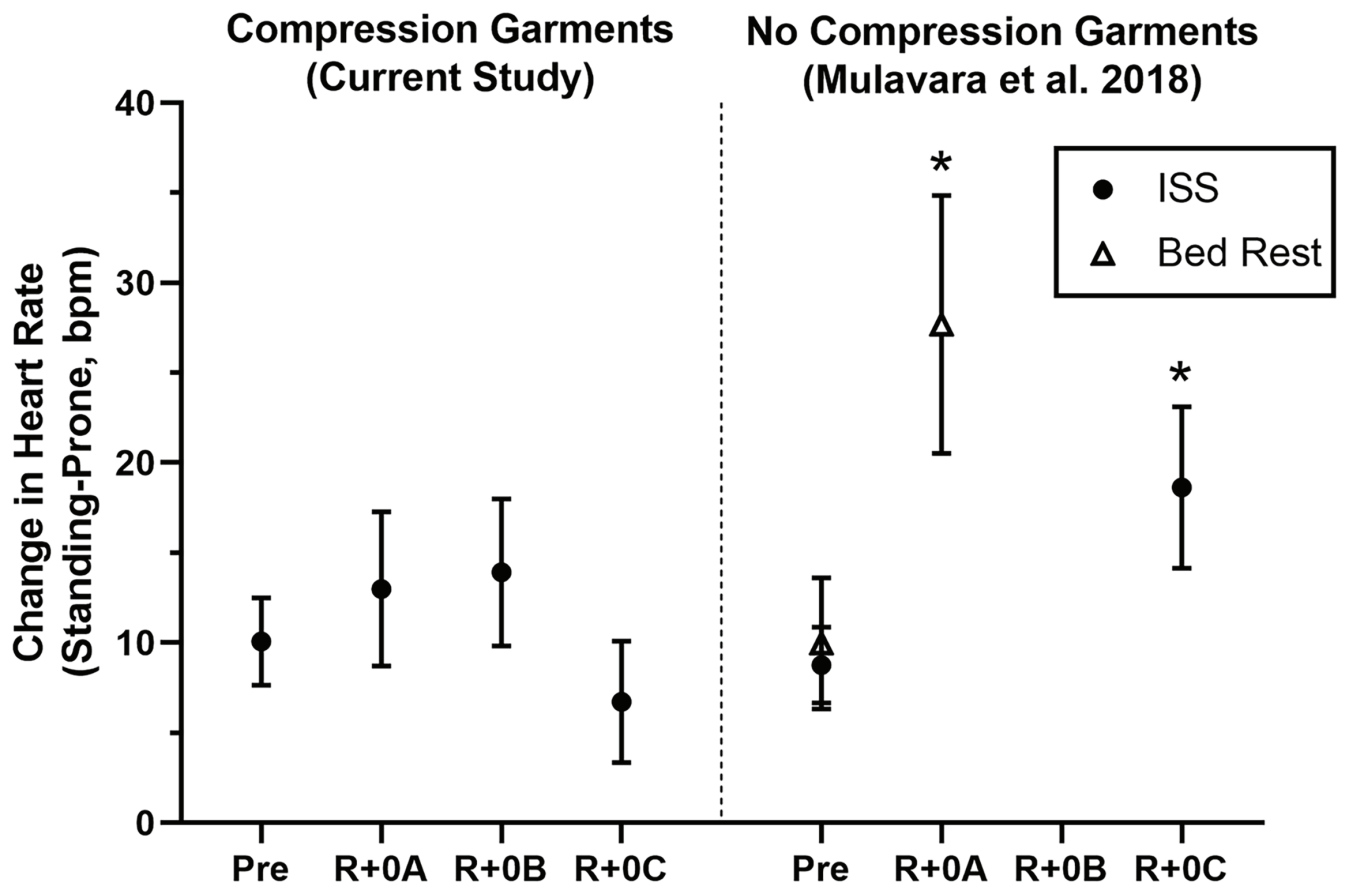
Mean change (±95% CI) in heart rate (Standing-Prone, bpm) in ISS astronauts (closed circles) participating in the current study who wore the GCG only during postflight tests (R+0A: 1.2–4.4 h; R+0B: 10.7–13.9 h; and R+0C: 25.6–30.7 h after landing) and in ISS astronauts (11 M, 2 F) and bed rest subjects (open triangles; 9 M) who participated in the same stand test protocol but did not wear compression garments (Mulavara et al., 2018). Results from these comparison groups of ISS astronauts and bed rest subjects are shown at time points comparable to the current study, and results were calculated in the same manner as in this study. ^*^Significantly greater preflight to postflight change in the heart rate response to standing than when wearing the GCG.

**Figure 2:**
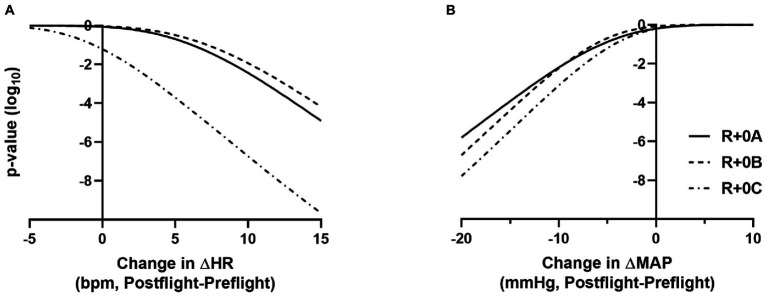
*p*-value functions (log_10_ metric) showing how well the data supports the mean preflight to postflight change in ΔHR exceeding N bpm, for −5 < N < 15 **(A)** and the mean preflight to postflight change in ΔMAP being lower than N mmHg, for −20 < N < +10 **(B)**. Plots are shown for R+0A, R+0B, and R+0C. For example, an actual mean preflight to postflight change in ΔHR exceeding 10 bpm at R+0A is not supported by the data (*p* < 10^−3^), and there is very little data support for a mean preflight to postflight change in ΔMAP being less than −15 mmHg at R+0A (*p* < 10^−4^).

Estimated means and 95% confidence intervals for heart rate and mean arterial pressure for prone rest, standing, and the change from prone to standing are shown in Table 1 for preflight and each postflight session. These results are consistent with the hypothesis that when wearing the GCG there would be no meaningful preflight to postflight mean change in either the ΔHR or ΔMAP from prone to standing. The mean (±SE) ΔHR before flight without the GCG was 10 ± 1 bpm (Figure 1), and the estimated mean differences from preflight ΔHR (95% CI) were +3 bpm (−2, 8) at R+0A, +4 bpm (−1, 9) at R+0B, and −3 bpm (−8, 1) at R+0C when wearing the GCG. Relative degree of data support (log_10_ p) for the preflight to postflight mean change of ΔHR exceeding hypothetical values ranging from −5 to +15 bpm is shown in Figure 2 for each postflight session. Compared with corresponding estimates of mean pre- to post-best or mean preflight to postflight change in ΔHR from Mulavara et al. (2018), the mean preflight to postflight change in ΔHR in astronauts wearing the GCG at R+0A was 15 bpm less than bed rest subjects not wearing the GCG [t(18) = −4.0, *p* = 0.0009] and 13 bpm less than ISS astronauts not wearing the GCG at R+0C [t(26) = −4.2, *p* = 0.0003].

**Table 1:** Estimated mean and 95% confidence intervals for heart rate and mean arterial pressure during prone rest (prone) and standing (stand) and the response to standing (Δ, stand-prone) before flight (preflight: mean of the two preflight tests, L-90 and L-60) and at R+0A (1.2–4.4 h after landing), R+0B (10.7–13.9 h after landing), and R+0C (25.6–30.7 h after landing). All means and confidence intervals were calculated with mixed-model linear regression analysis.

		Prone		Stand		Δ (Stand-prone)	
	*n*	Mean	95% CI	Mean	95% CI	Mean	95% CI
*Heart Rate* (*bpm*)							
Preflight	11	61	59, 64	71	68, 75	10	8, 13
R+0A	7	65	61, 68	78	71, 84	13	9, 17
R+0B	9	66	62, 69	80	74, 85	14	10, 18
R+0C	11	70	67, 72	76	71, 81	7	3, 10
*Mean Arterial Pressure* (*mmHg*)							
Preflight	11	91	88, 95	96	93, 100	5	3, 7
R+0A	7	102	94, 109	105	99, 112	3	-2, 9
R+0B	7	97	91, 103	97	88, 106	2	-3, 7
R+0C	11	97	92, 101	101	94, 107	4	-1, 8

In our ISS astronauts, the mean (±SE) ΔMAP from prone to standing before flight was 5 ± 1 mmHg when not wearing the GCG, and the estimated changes from preflight ΔMAP (95% CI) were −1 mmHg (−8, 5) at R+0A, −3 mmHg (−8, 3) at R+0B, and −1 mmHg (−6, 4) at R+0C when wearing the GCG. Relative degree of data support (log_10_ p) for mean change of ΔMAP being lower than hypothetical values ranging from −20 to +10 mmHg is shown in Figure 2 for each postflight session.

**Figure 3:**
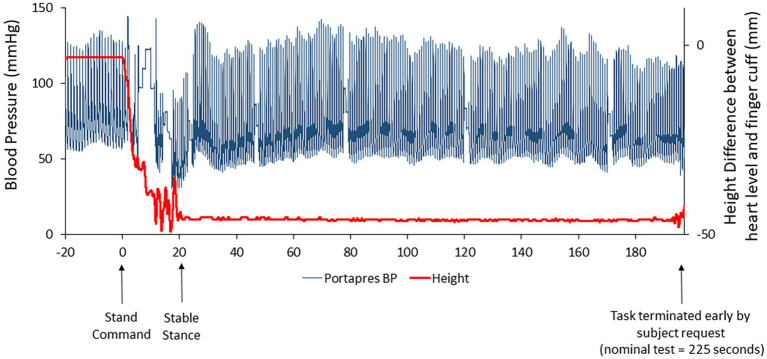
Beat-to-beat blood pressure tracing (raw data; blue line) in one subject who was unable to complete the stand test on R+0B. The red line represents the height correction sensor for the ambulatory blood pressure device (Portapres®) that was placed at heart level on the subject’s arm. The height correction is near “0” when the subject is prone but decreases when the subject is standing. Retrospective review of these data suggested that the subject was not hypotensive.

One subject was able to complete only 3.3 of the 3.5 min of standing at R+0B, requesting test termination due to apparent symptoms of motion sickness. At the end of the stand test, this astronaut reported a motion sickness score of 14 out of 20, the highest score reported for any subject participating in the stand test at R+0B. Retrospective review of the beat-to-beat blood pressure tracing revealed no indications of hypotension (Figure 3).

Of the 10 astronauts for whom IV and oral fluid data were recorded, nine reported oral ingestion of additional fluids in the 24 h before landing. Specifically, seven astronauts reported that they completed the protocol for end-of-mission fluid loading as prescribed by the Russian medical personnel, two reported ingesting 650–3,000 ml of fluid of unknown composition in addition to their normal consumption, and one did not report ingesting any additional fluid. Crew surgeon notes indicated that most astronauts received some IV fluids and all drank water or other beverages during the return to Houston. Of the data recorded by the crew surgeons, total IV fluid administration ranged from 300 to 4,000 ml, and oral fluid consumption ranged from 500 to 4,500 ml (Table 2). In these astronauts, many received anti-emetics before landing, and several received the same or similar medications during the first 25–30 h after landing (Table 3).

**Table 2:** Intravenous fluids administered by the flight surgeon and oral fluids ingested by the astronauts in the first 25–30 h after landing as recorded in the flight surgeon log. Prescribed: did the astronaut perform the end-of-mission fluid loading protocol as prescribed by the Russian medical personnel. NA, records not available; NR, none recorded.

	Fluid load		Kazakhstan		Midway		JSC	
	Prescribed	Amount (ml)	IV (ml)	Oral (ml)	IV (ml)	Oral (ml)	IV (ml)	Oral (ml)

1	Yes	NR	1000	400	1000	444	2000	2000
2	NA	NA	NA	NA	NA	NA	NA	NA
3	No	3000	1000	NR	1000	860	NR	500
4	NR	NR	NR	300	800	1500	NR	1850
5	Yes	NR	NR	500	1000	NR	NR	NR
6	Yes	NR	500	3500	NR	250	NR	750
7	Yes	900	2000	355	NR	355	NR	500
8	Yes	2600	1000	1000	NR	1000	NR	1000
9	Yes	NR	NR	473	NR	500	NR	937
10	Yes	NR	1000	NR	NR	944	NR	794
11	NR	650	300	NR	NR	NR	NR	NR

**Table 3:** Antiemetic medications received by the astronauts before landing (>6 h before R+0A), before R+0A (<6 h), and in the first 25–30 h after landing.

Subject	Before landing	Before R+0A	Kazakhstan to refueling	Refueling to JSC
1	Meclizine	Ondansetron, Promethazine	Meclizine	Promethazine
2	Not available	Not available	Not available	Not available
3	None reported	Promethazine	None reported	None reported
4	Ondansetron	None reported	Promethazine	None reported
5	None reported	Ondansetron, Promethazine, Meclizine	Promethazine	None reported
6	Scopalmine	None reported	Scopalamine	Scopalamine
7	Meclizine	Ondansetron	None reported	Meclizine
8	Meclizine	Meclizine, Ondansetron	None reported	None reported
9	Meclizine	Meclizine	None reported	None reported
10	Meclizine	Meclizine	None reported	None reported
11	None reported	None reported	None reported	None reported

## Discussion

Here we report for the first time that use of GCGs throughout the first 24 h after returning from long-duration spaceflight provides effective protection from the development of orthostatic intolerance during a brief stand test, extending our findings after short-duration spaceflight (Stenger et al., 2013). Given that the incidence of orthostatic intolerance is markedly increased after long-duration flight (Meck et al., 2001; Lee et al., 2015), and two astronauts in this study participated in missions greater than 270 days, these results suggest that use of the GCG can mitigate the risk of orthostatic intolerance after long-duration missions. However, astronauts demonstrating the most severe symptoms, such as nausea and/or dizziness, did not attempt to complete these tests, highlighting that integrated physiological responses are needed during re-acclimation to a gravitational environment. Even with the current suite of countermeasures in use (Kozlovskaya and Grigoriev, 2004; Lee et al., 2015; Loehr et al., 2015), not all astronauts will be tolerant of the upright posture during the period immediately following landing.

Although astronauts who did not wear the GCG were not tested as controls in this study, comparisons to similar data that have been previously published support the efficacy of the GCG as a countermeasure to orthostatic stress. In ISS astronauts who participated in early ISS missions (Expedition 1–17) and were tilted to 80° head-up without compression garments within 4 h of landing (*n* = 5), the average increase in heart rate from supine to 3 min of head-up tilt was ~25 bpm, resulting in a ~15 bpm higher standing heart rate than that measured during the same test before flight (Lee et al., 2015). These early ISS astronauts were prescribed exercise countermeasures using a similar philosophy by employing a combination of resistive and cardiovascular exercise (Lee et al., 2019), although the countermeasure hardware available during the early ISS missions was less robust than currently available (Korth, 2015; Loehr et al., 2015). Also, in bed rest subjects who performed an exercise countermeasures protocol similar to that used by ISS astronauts, the ΔHR from prone to standing in the same stand test protocol as used in this study increased from 7 bpm before bed rest to 25 bpm after bed rest (Figure 1; Mulavara et al., 2018). In contrast, in the current study of astronauts who completed the stand test in a similar timeframe, the mean change in heart rate from prone to standing at R+0A was only 13 bpm, which was only 3 bpm greater than preflight. From the *p*-value plots that we present here (Figure 2), the data did not support that when wearing the GCG after spaceflight the ΔHR from prone to standing will increase by more than 10 bpm during the stand test at R+0A. These findings are particularly important in our subjects given that six of the subjects tested at R+0A had not received IV fluids prior to testing at the landing zone in Kazakhstan, and therefore we expect that they would have been plasma volume depleted compared to their preflight condition (Meck et al., 2001). Our current findings were unchanged without the inclusion of the one astronaut who was tested at R+0A after receiving IV fluids.

There are no corresponding data against which to compare our R+0B data, but there are a few reports of orthostatic responses 1 day after landing (R+1), which is similar to the collection time of R+0C. In the astronauts participating in early ISS missions mentioned previously, the elevated ΔHR in response to tilt and elevated heart rate when tilted still were evident at R+1 (Lee et al., 2015). More recently, we reported that the ΔHR to the same stand test protocol as in the current study was 10 bpm greater on R+1 compared to preflight in ISS astronauts without the GCG, despite no difference in plasma volume between preflight and R+1 (Mulavara et al., 2018). In contrast, in the current study the ΔHR from prone to standing at R+0C was not different than preflight. However, Wood et al. (2019a,b) recently reported that the mean change in heart rate during a supine-sit-stand test, in which the stand portion was 3 min, did not change from preflight to postflight (preflight: 19; R+1: 21 bpm) in nine ISS astronauts, although the standing heart rate in those astronauts was significantly greater on R+1 (preflight: 75, R+1: 85 bpm). Together, results from the current study suggest that the GCG is an effective countermeasure to orthostatic stress on R+0.

There are potential limitations of comparisons of the current stand test results to other spaceflight studies, including that the duration of the stand portion of our protocol was shorter than some previous investigations during which presyncope was reported (Meck et al., 2001; Lee et al., 2015) and that the tilt test is considered to be more provocative. This stand duration was chosen such that failure to complete the stand test would not encourage the astronauts or flight surgeons to waive the remaining sensorimotor tests that were conducted in conjunction with this data collection (Mulavara et al., 2018). That mean arterial pressure was maintained in our subjects during standing when wearing the GCG is encouraging but results from our previous tilt test studies (Meck et al., 2001; Lee et al., 2015) suggest that this may have been an inadequate duration to observed decreases in blood pressure if the GCG was not effective.

An overall limitation of postflight studies of astronauts is that each crewmember is handled differently based upon their individual symptoms, the clinical judgment of the crew surgeon, and the conditions at the landing site. For example, in this study in some cases test sessions were waived or the protocol truncated due to the condition of the astronaut or the poor weather conditions at the landing zone (i.e., R+0A testing conducted at their airport in Kazakhstan instead of at the landing zone), and astronauts received different amounts of IV fluids or different medications. While these situations do not result in an ideal experimental design, they represent the actual conditions in which the data were collected and the range of conditions of ISS astronauts in the immediate post-landing period. Different number of subjects participated in postflight tests at R+0A and R+0B, which combined with our relatively small sample size, likely contributed to the width of the confidence intervals. Thus, we have attempted to document the conditions of each astronaut during testing to aid in the interpretation of these results.

Symptoms of motion sickness are common among astronauts after spaceflight (Jennings 1998) and influence tolerance to standing on landing day. In astronauts who are unable even to begin an orthostatic test, as we observed in this and our previous study (Lee et al., 2015), it is difficult to clearly ascribe a cause since there were no sensorimotor or cardiovascular data collected. Further complicating this matter is an interrelationship between the sensorimotor and cardiovascular dysfunction and the similarity of the symptoms, which might result in misclassification of the condition (Lackner, 2014). Appropriate monitoring of cardiovascular responses during sensorimotor challenges is required to differentiate the source of the symptoms. Astronauts often receive medication for motion sickness (Jennings, 1998; Shi et al., 2011), and in the 10 of 11 astronauts for whom we have at least partial reports from the flight surgeons, seven received at least one dosage of medication prophylactically before landing, six received medications soon before R+0A, five received medication in transit from Kazakhstan to the refueling stop, and three received medications between the refueling stop and arrival at JSC.

In particular, we were interested in examining information regarding astronauts who were unable to start the stand test at R+0A and R+0B as well as the one astronaut who started but was unable to complete the stand test at R+0B. Two of the three astronauts who were unable to begin the stand test at R+0A received at least one dose of Promethazine, either intramuscularly or intravenously, before testing was planned to begin. Although these two individuals likely were suffering from more significant sensorimotor disturbances, even if they had started the test they may not have been able to complete it. Promethazine is a H1-receptor antagonist that induces presyncope during orthostasis, primarily through an inhibition of sympathetic responses to protect blood pressure with no effect on heart rate (Shi et al., 2011). However, the one astronaut who was unable to start testing at R+0A and R+0B received only Meclizine and Zofran prior to R+0A, and no antiemetic medications were recorded before R+0B. Based upon notes received from the crew surgeon, the astronaut who started the stand test at R+0B but was unable to stand for the whole time, apparently suffered from symptoms of motion sickness throughout the 24-h period after landing. Symptoms included nausea and vomiting, and the individual received multiple dosages of Meclizine (25 mg, prophylactically), Zofran (4 mg SL), and Promethazine (12.5 mg IV) as well as normal saline IV (4 × 1 L).

Medications administered for motion sickness might have influenced our results at other times as well. For example, five of the seven astronauts had standing heart rates that were similar to or lower than the preflight value but two had standing heart rates that were more than 15 bpm higher at R+0A than preflight. Interestingly, mean arterial pressure was higher at R+0A than preflight in these two subjects, but these two individuals reported the highest motion sickness scores of the astronauts who completed testing at this time point. These two subjects also were the only ones who received Meclizine, another H1-receptor antagonist, ~30 min before testing, one of the potential side effects being sinus tachycardia.

Given that women have a higher incidence of postflight orthostatic intolerance than men (Fritsch-Yelle et al., 1996; Waters et al., 2002), it is important to assess whether the garments are effective for both sexes. While the number and proportion of women with spaceflight exposures were relatively low during the Space Shuttle (Harm et al., 2001) and early ISS programs, the number of women selected as astronauts and who have flown to space has steadily increased. Differences between sexes with regard to postflight orthostatic intolerance have been proposed to result from larger spaceflight-induced reductions in plasma volume in women coupled with a greater dependence on volume status (Waters et al., 2002) and heart rate responses (Gotshall et al., 1991; Frey et al., 1994), lower vascular resistance (Waters et al., 2002), and a smaller, less compliant left ventricle (Fu et al., 2004). Unfortunately, no women wore lower body compression garments in our previous study of Shuttle astronauts after short-duration spaceflight (Stenger et al., 2010, 2013), but four of the 16 subjects studied in a 14-day bed rest study were women, and no subjects became presyncopal when wearing the GCG during a 15-min 80° head-up tilt test on the last day of bed rest (Stenger et al., 2014). In the current study, two of 11 ISS astronauts were women, the ΔHR from prone to standing after spaceflight was similar or lower than preflight, and neither experienced hypotensive responses during the stand test while wearing the GCG.

We have previously reported that the incidence of presyncope during orthostatic tests dramatically decreases in the days after landing (Meck et al., 2001; Lee et al., 2015; Mulavara et al., 2018), yet some crewmembers are still intolerant of the upright posture or become hypotensive while standing during the days after return to Earth. Thus, wearing compression garments for several days after landing is warranted in some individuals. For example, we (Lee et al., 2015) previously reported that one ISS astronaut was unable even to start the tilt test on R+1, and two astronauts failed to complete the entire 10 min of tilt on R+3. Further, Wood et al. (2019a) reported that two astronauts (of 9 M astronauts) became hypotensive during a 3-min stand test conducted 18–36 h after landing. However, Fu et al. (2019) observed no hypotensive events using ambulatory blood pressure recordings acquired in 12 ISS astronauts, including four women, when participating in activities of daily living in the first 24 h after landing. Interpretation of ambulatory data is complicated because the authors had insufficient information to determine when the astronauts were wearing the Russian Kentavr or were supine during the postflight period. The plane transporting the astronauts back from Kazakhstan is equipped with a bed for each astronaut, which would be well-utilized if the individual suffered from sensorimotor disturbances, and many astronauts continue to wear the Kentavr or other compression garments.

Our data highlight the efficacy of the GCG during standing in the postflight period but the GCG has not been tested or relied upon during re-entry and landing. In the Soyuz and the currently planned configuration for the Orion capsule, the vast majority of the acceleration is directed anterior-posterior (G_x_) such that the head-to-foot (G_z_) stress is minimized and the likelihood of acceleration-related hypotension is minimized. The benefits of astronauts wearing compression garments during re-entry and landing would be more pronounced in vehicles that return from space with the subjects in position such that the acceleration vector is directed from the head to the foot, as was the situation during Space Shuttle landings. Though lower body compression garments were shown to be efficacious during re-entry of the Space Shuttle (Perez et al., 2003), NASA and the Russian Space Agency have no immediate plans for future space vehicles traveling to and from ISS in which the acceleration would be experienced by astronauts in G_z_. However, NASA plans to return to the moon by 2024 in a space vehicle that may include G_z_ accelerations with the astronauts standing, as they did during the Apollo missions. Not providing a seat for lunar descent and ascent reduces mass and volume requirements for the space capsule. There were no reports of hypotension during lunar descent and ascent, but the Apollo astronauts all were men and at least partially selected based upon their ability to tolerate sustained G-forces. Plasma volume (Leach et al., 1996) and the ability to tolerate orthostatic stress rapidly declines (Nixon et al., 1979; Bungo and Johnson, 1983) in the first few days of spaceflight, and thus some individuals might not be able to tolerate the G_z_ accelerations during descent to and ascent from the lunar surface. For example, women are more likely to experience orthostatic intolerance during re-exposure to gravity (Fritsch-Yelle et al., 1996; Waters et al., 2002), even at levels less than 1-G_z_. Grenon et al. (2006) reported 50% of women could not complete a 10-min 30° head-up tilt test after 2 weeks of bed rest. This tilt angle approximates the orthostatic stress equivalent to 0.5-G_z_, which is in the range of G_z_ experienced by Apollo astronauts during lunar descent and ascent. It is likely that a lower body compression garment like the GCG would be helpful in this situation.

## Conclusion

Wearing a garment that provides a gradient compression from the feet to over the abdomen after long-duration spaceflight prevented the tachycardia that normally occurs while standing after spaceflight without compression garments and protected against a decrease in blood pressure during a short stand test. A GCG would be an efficacious countermeasure to orthostatic intolerance during re-entry and landing and would provide orthostatic support during the reconditioning period.

## Data Availability Statement

Given the unique nature and size of our subject population, it is not possible to make these data publicly-available without compromising subject confidentiality and privacy. Requests to access the datasets should be directed to NASA’s Life Sciences Data Archive (https://lsda.jsc.nasa.gov/).

## Ethics Statement

The studies involving human participants were reviewed and approved by NASA Johnson Space Center Institutional Review Board, Institute of Biomedical Problems Bioethics Committee, and the ISS Human Research Multilateral Review Board. The participants provided their written informed consent to participate in this study.

## Author Contributions

SML contributed to the study design, implementation, interpretation of results, drafting and revision of the manuscript, and approval of final draft. LR contributed to the study implementation, editing of the manuscript, and approval of final draft. SSL contributed to the interpretation of results, editing of the manuscript, and approval of final draft. AF contributed to the statistical analyses, drafting and editing of the manuscript, and approval of final draft. VK contributed to the study implementation and approval of final draft. IK contributed to the study implementation, editing of the manuscript, and approval of final draft. BM contributed to the interpretation of results, editing of the manuscript, and approval of final draft. MR contributed to the study implementation, editing of the manuscript, and approval of final draft. IR contributed to the study implementation and approval of final draft. ET contributed to the study design, implementation, editing of the manuscript, and approval of final manuscript. JB contributed to the study design, implementation, interpretation of results, editing of the manuscript, and approval of final draft. IK contributed to the study design, implementation, editing of the manuscript, approval of final draft, and secured funding. MFR contributed to the study design, implementation, interpretation of results, editing of the manuscript, approval of final draft, and secured funding. MS contributed to the study design, implementation, interpretation of results, editing of the manuscript, approval of final draft, and secured funding. All authors contributed to the article and approved the submitted version.

## Conflict of Interest

SML, LR, SSL, IK, BM, and MR were employed by KBR, under the Human Health and Performance Contract to NASA, during the performance of this study.

The remaining authors declare that the research was conducted in the absence of any commercial or financial relationships that could be construed as a potential conflict of interest.
